# Comparison of anger management, anxiety and perceived stress in patients with cancer and Coronary Heart Disease (CHD)

**Published:** 2015

**Authors:** M Aghaei, N Ghorbani, R Rostami, A Mahdavi

**Affiliations:** * Department of Psychology, Faculty of Psychology and Educational Sciences, University of Tehran, Tehran, Iran; ** Department of Psychology, Faculty of Psychology and Educational Sciences, University of Payame Noor, Tehran, Iran

**Keywords:** anger management, anxiety, perceived stress, patients with cancer and CHD

## Abstract

The present study was conducted with the aim of studying the comparison of anger management, anxiety and perceived stress in patients with cancer and Coronary Heart Disease (CHD). The two groups of subjects consisted of patients with cancer (n = 120) and patients with CHD (n = 118) who were selected by using a convenience sampling method and by the employment of Spielberger’s State-Trait Anger Expression, Costello and Camry Depression and Anxiety scale and Perceived Stress Scale of Cohen, Kamarak and Mermelstein. In order to analyze the data, after the calculation of descriptive statistics and correlation coefficients, MONOVA was employed to test the hypotheses. The findings indicated that patients with CHD regulate the excitement by externalizing anger and patients with cancer control this excitement by internalizing anger. Moreover, stress and depression in patients with CHD were greater than in patients with cancer. The findings were explained by the employment of the theoretical patterns of the mediating role of personality and regulation of excitement in stress and illness.

## Introduction

Thoughts and feelings have a great impact on the physical health of people. Physical diseases such as stomach ulcers, coronary heart disease, cancer, and asthma are physical reactions that can be produced by thoughts and feelings. Many evidences showed that the duration and even the occurrence of such a disease could be influenced by the psychological status of the patients [**1**]. Among the psychological factors, the impacts of negative excitements such as anxiety, depression, anger, and the method of regulation (externalizing anger and internalizing anger) were studied in comprehensive physical disorders such as CHD and cancer [**2**]. Generally, thrilling experiences are regulated in three ways: repression, suppression, and expression [**3**].

Research evidences confirmed that chronic anxiety increases the risk of coronary heart disease through A) effects on unhealthy behaviors (e.g. smoking), B) increase of atherosclerosis (means: increase of hypertension) and C) heart problems, including irregular heartbeat or accelerated thrombosis [**4**]. In addition, there was a relationship between depression and myocardial infarction [**3**].

One of the most insidious diseases that is influenced by psychological factors is cancer [**1**] and a significant relationship between depression and the cancer disease has been frequently reported in clinical studies [**5**]. Although severe and chronic depression might increase the risk of cancer, there have been limited evidences about the mortality rate of cancer due to depression. One of the assumptions is the lack of coordination between the psycho physiological mechanisms of the relationship of depression and the cancer progression based on Hypothalamus Pituitary Adrenal (HPA) and depression, which might directly affect the immune system [**6**]. Many factors such as the high level of stress, experiencing psychological issues and having a psychiatric diagnosis were considered in a study on the relationship between the negative excitements and the growth of cancer. According to the emotional expression and inhibition in cancer patients, it was shown that the more expression and less inhibition of emotions predict a more longevity in cancer patients [**7**]. The findings indicated that externalizing anger in patients with cancer is much more common than in control groups [**8**]. Based on the mentioned findings, the present research validated two destructive methods of externalizing and internalizing anger in two groups of patients with cancer and patients with CHD, and their relationship in the meantime. Compared to the patients with cancer, it was predicted that patients with CHD regulate this excitement by externalizing anger while compared to patients with CHD, patients with cancer usually regulate this excitement by internalizing anger. In addition, the following questions were answered: Were there any differences between the amounts of stress in the two groups? Was there more depression in patients with cancer compared to patients with CHD?

**Methodology**

The present research was of causal-comparative type or post-event research. In order to implement the research, after the necessary arrangements with Shariati and Imam Khomeini hospitals, the questionnaire that consisted of the scales of anger, anxiety, and stress, respectively, was conducted individually on the subjects and in a self-assessment manner. The population consisted of all the patients with cancer and CHD who referred to the hospitals. The sample of patients with cancer was obtained from the patients who were admitted to the surgical ward of cancer and were referred and admitted to the central sections 1 and 2 of Imam Khomeini hospital for chemotherapy. The sample of patients with CHD was obtained from the patients in the sections of CCU, surgery, and post cat. In the meantime, 120 of the cancer patients were admitted to the surgical ward of cancer, and were referred and hospitalized in the central part 1 and 2 of Imam Khomeini Hospital, and 118 of the patients who were admitted to the sections of CCU post cat or cat lab and cancer surgery were selected as a sample size by using the convenience sampling method. In order analyze the data, MONOVA was employed to test the hypotheses after the calculation of the descriptive statistics and the correlation coefficients.

**Research tools**

1) State-Trait Anger Expression (STAXI-2)

This questionnaire was developed by Spielberger CD, Johnson EH, Russell SF, Crane RJ, Jacobs GA, Worden TJ [**9**] in order to study the way of expressing anger and was revised in 1990. The revised version of the questionnaire was employed in the present research that consisted of 6 main scales and 5 sub-scales in order to investigate experience, expression and control of anger. In the revised scale, 8 items were added to evaluate anger control in two fields of control in and control out. The revised scale consisted of 57 items and each item had 4 options and the subjects graded themselves by using a 4-point Likert scale (from 1 - almost never to 4 - usually). The scale of the state of anger and its sub-scales were not used in the present research and the scale of the trait anger and two of its sub-scales, including Trait Angry Temperament and Trait Angry Reaction and scales of externalizing anger, internalizing anger, internal control of anger, and external control of anger, were used in the present research. The scale of trait anger was used to evaluate the way of experiencing anger. This scale had two sub-scales and 10 items. The reliability coefficient of the general trait of anger was obtained as being equal to 0.85 for women and 0.86 for men, by using Cronbach’s alpha method. The scale of externalizing anger was used for evaluating the way of expressing anger physically or verbally. This scale also had 8 items and its reliability coefficient was 0.75 for women and 0.73 for men. The scale of internalizing anger was used to evaluate the way of experiencing anger without expressing it. This scale consisted of 8 items and its reliability coefficient was 0.78 for women and 0.74 for men. The scale of external control of anger was used to evaluate the way of controlling anger externally. This scale had 8 items and its reliability coefficient was 0.85 for women and 0.83 for men. The scale of internal control of anger was used to evaluate the way of controlling anger by using staying calm. This scale had 8 items and its reliability coefficient was 0.93 for women and 0.91 for men. Spielberger and his co-workers presented two overall reliability coefficients for the total scales and subscales for both sexes, whose reliability coefficient was 0.76 for men and women. Spielberger also implemented the questionnaire on 1900 individuals to evaluate normal and abnormal individuals. The internal consistency of the scales and sub-scales in normal and abnormal individuals was 0.73 and 0.95, respectively (Spielberger et al., 1998). The questionnaire was first translated and the translation was given to the experts and scholars in this field. After the confirmation of the translation, the questionnaire was implemented on the patients.

2) Depression and Anxiety scale

This scale had 9 items for anxiety and 14 items for depression, which was validated by Ghorbani N, Davison H, Watson PJ, Bing NM, Maek AD [10] and by using Cronbach’s alpha method, the coefficients being 0.74 and 0.84 for anxiety and depression, respectively.

3) Perceived Stress Scale

This scale was also validated by Ghorbani N, Davison H, Watson PJ, Bing NM, Maek AD [**10**] and by using Cronbach’s alpha method, the coefficients being equal to 0.81.

**Findings**

The average age of the patients with cancer was 39.5 and the standard deviation was 9.61. The values were 52.2 and 9.61 respectively, in the patients with CHD. The education levels in both groups were high school education. More than 25% had high school education and in the groups of patients with cancer and CHD, 46.7% and 44.1% had a high school diploma, 10% and 13.6% had an Associate Degree, 17.5% and 13.6% had a bachelor degree and 0.83% and 3.4% had a master degree and more. In terms of marital status, 82.5% of the patients with cancer were married, this representing 98.3% for the patients with CHD. Accordingly, 17.5% of the patients with cancer were bachelor and 1.6% of the patients with CHD were bachelor.

**Table 1** shows the correlation between the variables in the two groups. As it was obvious, in the samples with cancer, the correlation between internalizing anger and depression was 0.039 and in samples with CHD, it was 0.26, while the correlations between externalizing anger and depression were 0.08 and 0.21 in the samples with cancer and in the samples with CHD, respectively.

The results of the data analysis and testing of research hypotheses indicated that there was a significant difference (P < 0.000 and F = 10.07). The significant findings were in line with the research hypotheses, which are listed in **Table 2**. In the investigation of the first hypothesis, it could be expressed that in comparison with the patients with cancer, patients with CHD regulated the excitement by using externalizing anger. The average scores for anger in women patients with CHD, and men patients with CHD were 2.27 and 2.26, respectively, which were greater than the scores for patients with cancer (1.76 and 1.96 for women and men, respectively). In other words, compared to the patients with cancer, when dealing with anger, patients with CHD externalized it. The findings of the second and third hypotheses were the following:

The average score of internalizing anger in women patients with cancer (2.18) and men patients with cancer (2.17) were greater than the one of women patients with CHD (1.85) and men patients with CHD (1.90) (P < 0.05 and F = 2.80). In other words, compared to patients with CHD, when dealing with anger, patients with cancer mostly internalized them. As it was clear, sex had no impact in the meantime.

According to the findings, the average scores of stress among women patients with CHD (3.32) and men patients with CHD (2.87) were greater than the scores of women patients with CHD (2.82) and men patients with CHD (2.39) (P < 0.001 and F = 25.06), therefore, it could be deduced that stress was higher in patients with CHD.

The findings in the table below indicated that the average scores of depression in women patients with CHD (2.86) and in men patients with CHD (2.41) were greater than the scores of women with cancer (2.40) and the men patients with cancer (2.39) (P < 0.21 and F = 5.39). In other words, the depression levels of patients with cancer were not higher in patients with CHD.

**Table 1:** Correlation matrix of the research variable in the cancer and CHD samples

Variable	1	2	3	4	5	6	7	8	9	10
General trait of anger	-	0.64**	0.38**	-0.29**	0.17	0.17	0.14	0.33**	0.15	0.31**
Trait anger temperature	0.844**	-	0.41**	-0.20**	-0.23	0.26**	0.21*	0.31**	0.30**	0.37**
Trait anger reaction	0.62**	0.17	-	-0.43**	0.22*	0.17	0.54**	0.63**	0.45**	0.60**
Externalizing anger	0.44*	0.20*	0.47**	-	0.63**	0.11	-0.43**	0.49**	-0.21*	0.41**
Internalizing anger	0.29	0.27	0.14	0.11	-	0.10	-0.43**	0.49**	-0.21*	0.41**
Internal control of anger	-0.23*	-0.11	-0.27**	-0.0008	-0.30**	-	0.09	0.20*	0.19*	0.25**
External control of anger	0.24**	-0.42	-0.38**	-0.12	-0.20*	0.69**	-	0.67**	0.52**	0.72**
Anxiety	0.43**	0.25**	0.45**	0.23**	0.40**	-0.47**	-0.39**	-	0.47**	0.84**
Depression	0.30**	0.17	0.29**	0.08	-0.40**	-0.25**	-0.36**	-	0.83**	
Perceived stress	0.29**	0.17	0.26**	-0.04	0.24**	-0.44**	-0.28**	0.60**	0.64**	-

The results related to the subjects with cancer were above the diagonal line and the results related to the subjects with CHD were under the line.

*Significance level P < 0.05

**Significance level P < 0.001

**Table 2:** Correlation between subjects’ impact of variables in the two groups

Group->	Women with cancer		Men with cancer		Women with CHD		Men with CHD		F Statistic	Sig. level
↓Variable	Average	Standard deviation	Average	Standard deviation	Average	Standard deviation	Average	Standard deviation		
General trait of anger	1.72	0.35	1.80	0.45	2.43	0.52	2.33	0.61	67.19	0.001
Trait anger temperature	2.27	0.69	2.22	0.72	2.72	0.69	2.26	0.77	14.94	0.001
Trait anger reaction	1.41	0.38	1.58	0.47	2.67	0.68	2.35	0.73	129.78	0.001
Externalizing anger	1.76	0.32	1.92	0.42	2.27	0.37	2.26	0.52	41.37	0.001
Internalizing anger	2.18	0.43	2.17	0.47	1.85	0.35	1.90	0.10	2.80	0.05
Internal control of anger	3.03	0.66	3.18	0.71	2.72	0.47	2.81	0.53	15.78	0.001
External control of anger	3.20	0.66	3.22	0.51	2.46	0.46	2.70	0.59	51.29	0.001
Anxiety	2.86	0.71	2.64	0.78	3.75	0.73	3.31	0.79	45.63	0.001
Depression	2.40	0.89	2.30	0.82	2.76	1.05	2.41	0.70	5.39	0.21
Perceived stress	2.82	0.70	2.39	0.70	3.32	0.74	2.87	0.54	25.06	0.001

## Conclusion

As considered in the findings, patients with CHD externalized their anger compared to patients with cancer. The emotional disturbance had a close relationship with an increase in death and severity of illness in patients with CHD. In fact, emotional disturbance prompted pathophysiological mechanisms and these mechanisms led to an increase in Coronary Vascular Constriction and consequently, an increase in the activities of plaques, which had a significant impact in the elevation of death in CHD [**11**]. The findings were in line with the other similar researchers such as the ones of Kabzansky LD, Kawachi I (2000), Kabzansky LD, Kawachi I. Weiss ST, Sparrow D (1998), Oppels A (1997), Siegman AW, Townsend S, Blumenthal RS, Sorkin JD, Cahid Civelek A (1998), Spielberger CD, Reheiser EC, Sideman SJ (1995) [**3**,**4**,**12**-**14**].

The research results demonstrated that compared to patients with CHD, in dealing with anger, patients with cancer mostly internalized it. The findings were in line with the results of Garssen B (2004), Burns JW (1997), Grossarth–Maticek R, Siegrist J, Vetter H (1982), Greer S, Morris T (1987) [**7**,**8**,**15**,**16**]. Moreover, some of the studies indicated that the experimental induction of individuals to writing or talking about their stressful experiences in some sessions could lead to the physical and physiological health of the patients with cancer. Smith’s analysis about 13 researches conducted on emotional revelations indicated that 23% of the improvement occurred in the experimental group which had emotional revelations [**2**,**17**]. On the other hand, patients with self-controlled character endured a more psychological pressure and this led to the growth of cancer mediated by the immune system (Rainbow et al., 2004). Patients with cancer seemed to have had more needs to express their negative emotions compared to normal individuals. However, they had a tendency to control their emotions or feelings and researches demonstrated that victims of cancer were not effective in the formation and maintenance of long-term close relationships with others. This resulted in the reduction of the social protection in these patients, which was accompanied by a health risk in patients. In addition, women who were classified as consistent showed indications of learned helplessness and therefore seemed to be more consistent. It had been seen that the activities of natural killer cells were less but necessary [**18**].

The research findings showed that the stress level was higher in patients with cancer. The result of the research was in line with the ones of Siegman AW, Townsend S, Blumenthal RS, Sorkin JD, Cahid Civelek A (1998), Bruehl S, Chung C, Burns JW (2003) [**13**,**19**]. The second section of the findings of the hypothesis was that patients with cancer had less stress compared to the patients with CHD. The findings were not in line with the results of researches such as Brannon & Linda (1997) [**20**].

The study of the results indicated that patients with CHD had higher levels of depression than patients with cancer. This rejected the present research hypothesis, which expressed that depression in cancer patients was higher. The finding was in line with some of the previous researches and it was not in line with some others. For instance, it was in line with the results obtained from Ho RTH, Chaw CLW, Ho SMY (2004) [**21**], and it was not in line with the results of Shemel & Iker (1971), Biliaskaz and Garon, cited in Ho RTH, Chaw CLW, Ho SMY (2004) [**21**]. It seemed that a high depression in patients with CHD resulted from the destruction of intimate relationships caused by the externalization of anger. It should be noted that there was a high correlation between depression and the internalization of anger in patients with cancer and the higher depression and externalization in patients with CHD. This was in line with the mentioned explanation, hence, the different reasons for the depression in patients with cancer and CHD should be paid a special attention. It seemed that according to the imminence of a sudden death from heart attack, the higher stress levels of these patients with respect to patients with cancer could be explained. The present research results were in line with the results obtained from the researches of Spielberger CD, Johnson EH, Russell SF, Crane RJ, Jacobs GA, Worden TJ (1985), Spielberger CD, Reheiser EC, Sideman SJ (1995), and Thomas SP (1989) [**9**,**14**,**22**].
